# Supplementary material for: Enhanced surface colonisation and competition during bacterial adaptation to a fungus
Source: Nat Commun. 2024 May 27;15:4486. doi: 10.1038/s41467-024-48812-1 (PMC11130161; doi:10.1038/s41467-024-48812-1)
Supplement: Supplementary file 1 — Supplementary Information [file 41467_2024_48812_MOESM1_ESM.pdf]

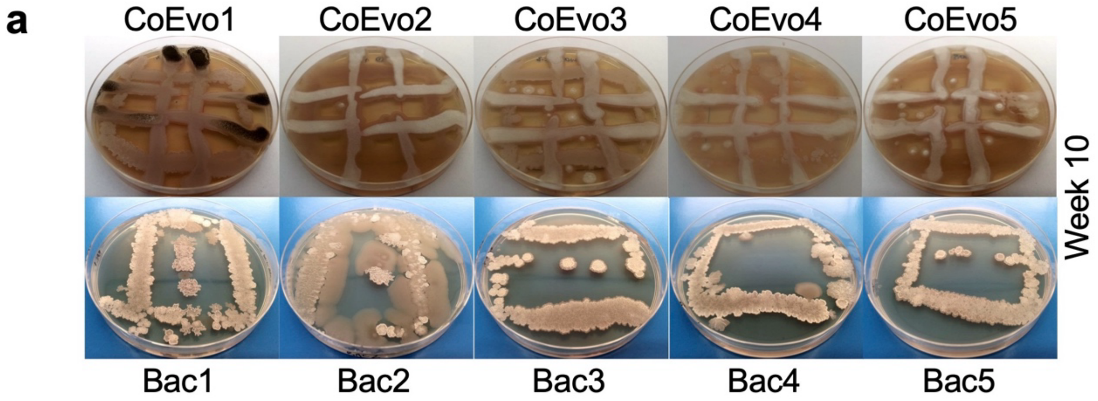

Supplementary figure 1 | *B. subtilis* adaptation to the presence of *A. niger*. a, Experimental evolution plates at the 10th transfer, top panels showing *B. subtilis* evolved in the presence of *A. niger*, and lower panels showing bacteria only cultivations. The plate size = 9 cm, CoEvo refers to co-culture evolved isolates, Bac denotes bacteria only evolved isolated.

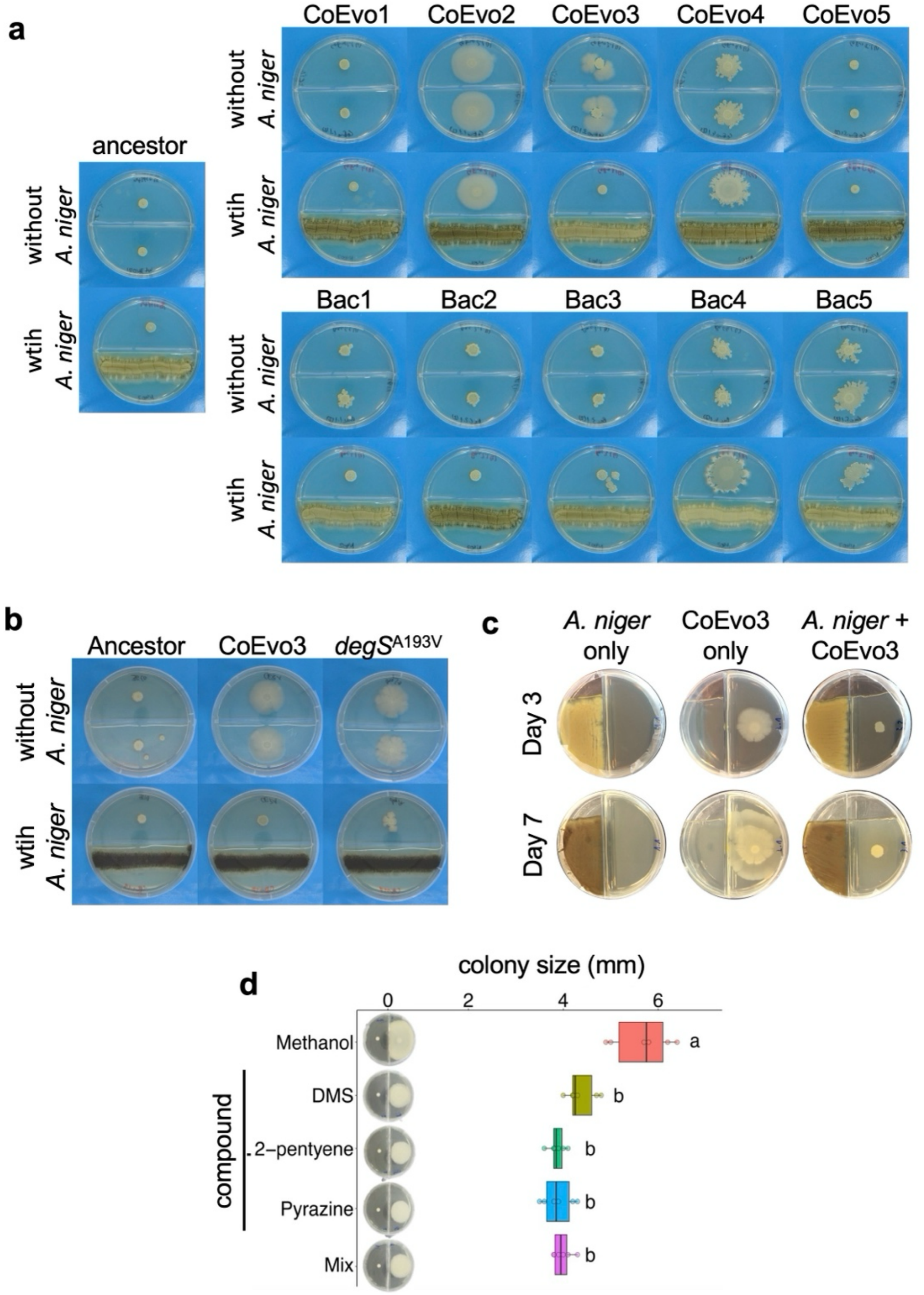

Supplementary figure 2 | The effects of volatile compounds on *B. subtilis* growth.

a, Colony spreading of the ancestor and evolved isolates in the absence (top panels) and presence of *A. niger* (lower panels). **b,** Colony spreading of the ancestor, CoEvo3 and *degS*^{A193V} mutant in the absence (top panels) and presence of *A. niger* (lower panels). **c,** Experimental setup used to trap VOCs at day 3 and 7. The empty space in the agar medium was used to place the steel traps containing 150 mg Tenax TA and 150 mg Carbopack B. For panel a, b and c, representative images are shown from at least 3 repeats. **d,** Colony spreading of the CoEvo3 isolate recorded in the presence of methanol, dimethyl disulphide (DMS), 1-pentyne, pyrazine, or their mixture. One-way ANOVA was conducted to compare the effects of pure compounds and the mixture on CoEvo3 colony size. The results indicate a statistically significant difference in colony size between methanol treatment and other treatments ($F = 27.04$, $p < 0.001$, $n = 6$). Tukey's HSD test was used to determine significant differences between means, which are denoted by letters.

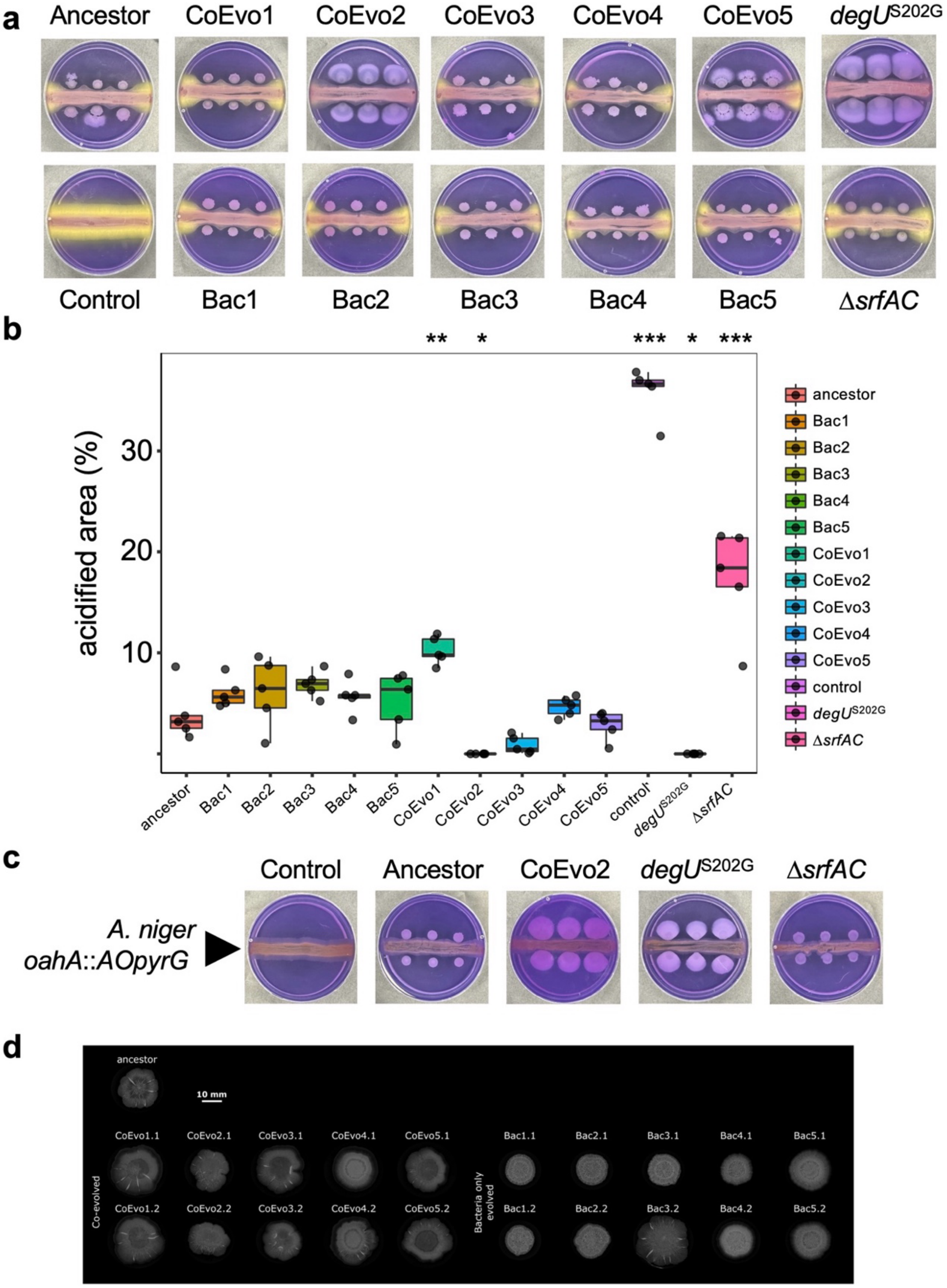

Supplementary figure 3 | *A. niger*- mediated acidification and *B. subtilis* colony biofilms. a, Panels are showing the pH of the medium using Bromocresol Purple, where bacterial cultures were spotted next to a continuous fungal spore streak. Purple and yellow colours indicate pH >6.8 and pH <5.2, respectively. The plate size = 9 cm, CoEvo refers to co-culture evolved isolates, Bac denotes bacteria only evolved isolated. Control includes fungal inoculation in the absence of *B. subtilis*. One representative image is shown from 5 repeats. **b,** Quantitative measurement of relative acidification area of using Image J, measuring the area according to colour thresholds specific to the yellow and dividing by the area of the whole plate (see materials and methods). Statistically significant differences were examined using one-way analysis of variance (ANOVA) followed by Tukey's multiple comparisons with the ancestor (*, P <0.05; **, P < 0.01; ***, P < 0.001, n = 5). **c,** Images show the non-acidifying *A. niger oahA*::*AOpyrG* strain and respective *B. subtilis* strains inoculated on pH indicator plates as in panel a. The plate size = 9 cm. One representative image is shown from 3 repeats. **d,** Biofilm colonies of evolved isolates on MSgg agar medium. Scale bar = 10 mm.

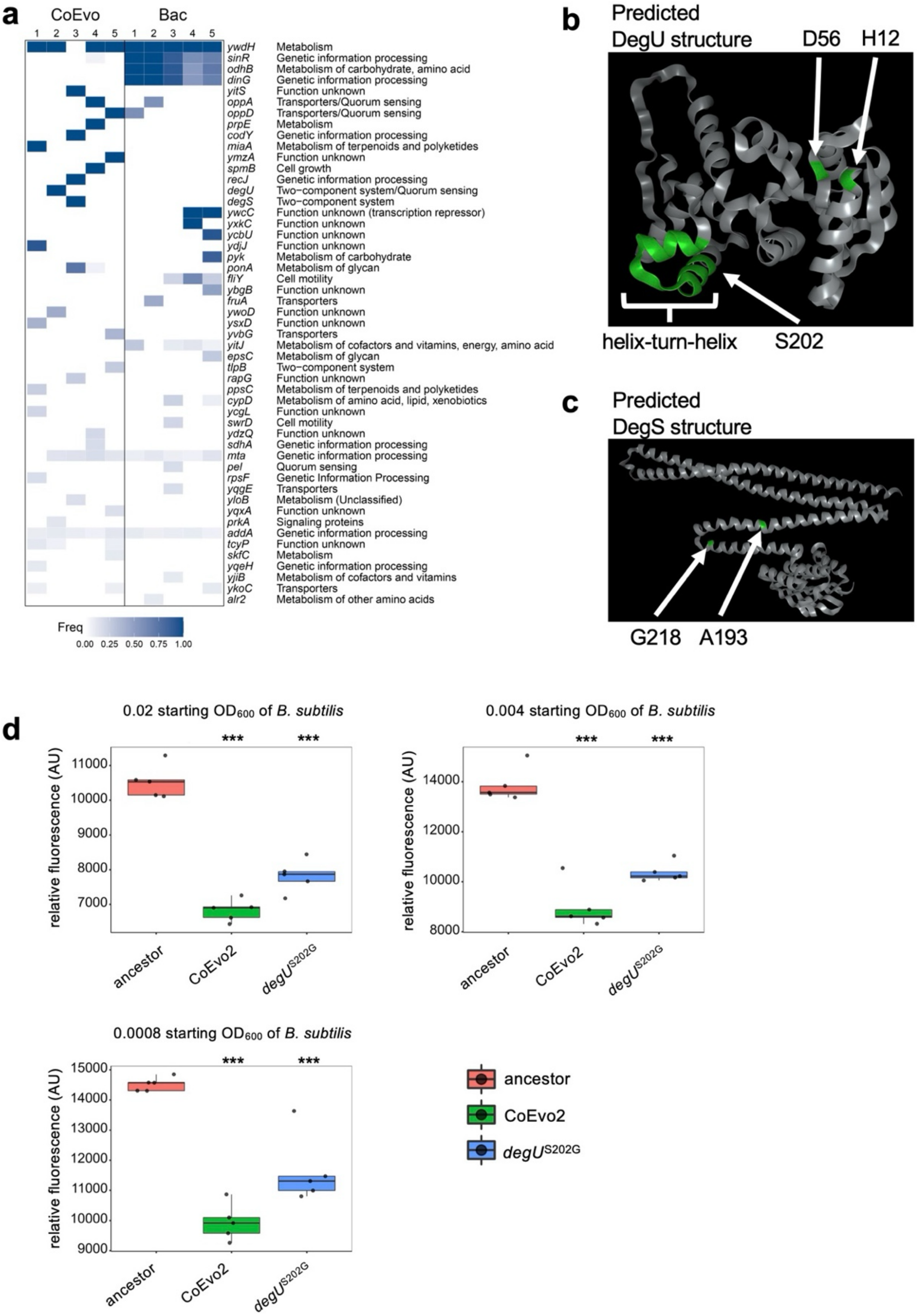

Supplementary figure 4 | Genetic characterisation of *B. subtilis* adaptation to *A. niger*. a, Detected mutations in CoEvo and Bac populations. **b,** Predicted DegU structure based on AlphaFold (https://alphafold.ebi.ac.uk/entry/P13800). H12 and D56 amino acids are highlighted that were previously described to be involved in phosphorylation state of DegU. SNP in CoEvo2, S202 is also highlighted. **c,** Predicted DegS structure based on AlphaFold (https://alphafold.ebi.ac.uk/entry/P13799). G218 amino acid is highlighted that were previously described to be involved in phosphor-activity of DegS. SNP in CoEvo3, A193 is also highlighted. **d,** Growth of the *A. niger* P_{gpdA}-sGFP-T*trpC* after 72 hours detected using measuring the constitutively produced GFP in the presence of different starting OD_{600} bacterial cultures of ancestor, CoEvo2, and *degU*^{S202G} strains. Different starting OD_{600} of the bacterial culture was used according to the previously (see Materials and Methods). Statistically significant differences were examined using one-way analysis of variance (ANOVA) followed by Tukey's multiple comparisons with the ancestor ***, $P < 0.001$, n=5).

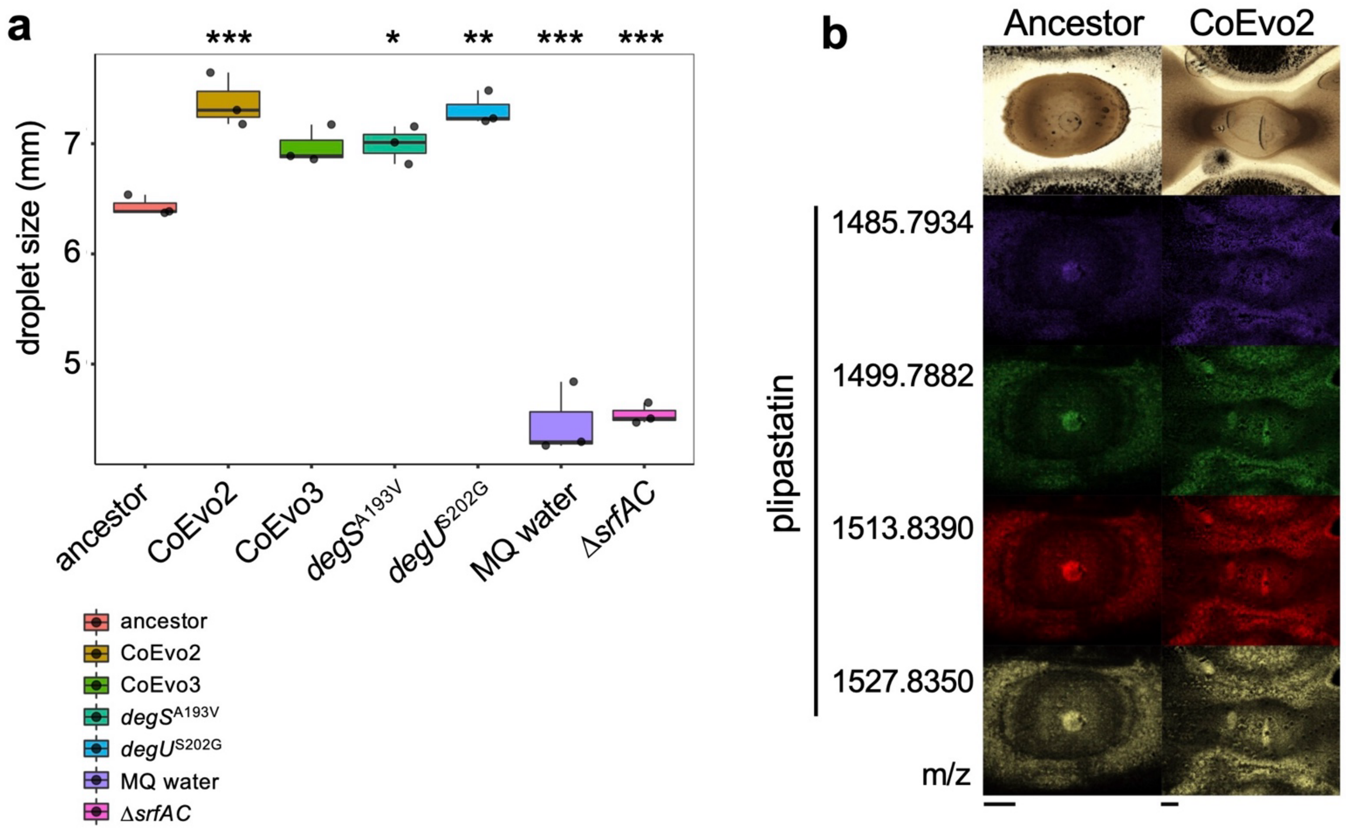

Supplementary figure 5 | Semi-quantitative detection of surfactin production and spatial detection of plipastatin in CoEvo2. a, The size of droplets on parafilm of bacterial supernatants that correlates with the amount of surfactin produced. Droplet size was measured using Image J and compared to a ruler. Statistically significant differences were examined using one-way analysis of variance (ANOVA) followed by Tukey's multiple comparisons with the ancestor (*, P <0.05; **, P < 0.01; ***, P < 0.001, n=3). CoEvo3 P = 0,0500435. **b,** MALDI-MSI spatial detection of plipastatin isoforms in bacterial colonies (wild-type and CoEvo2) grown between two fungal streak lines. m/z values of surfactin isoforms are indicated on the left. Scale bars = 2 mm. MALDI-MSI experiments have been performed twice.

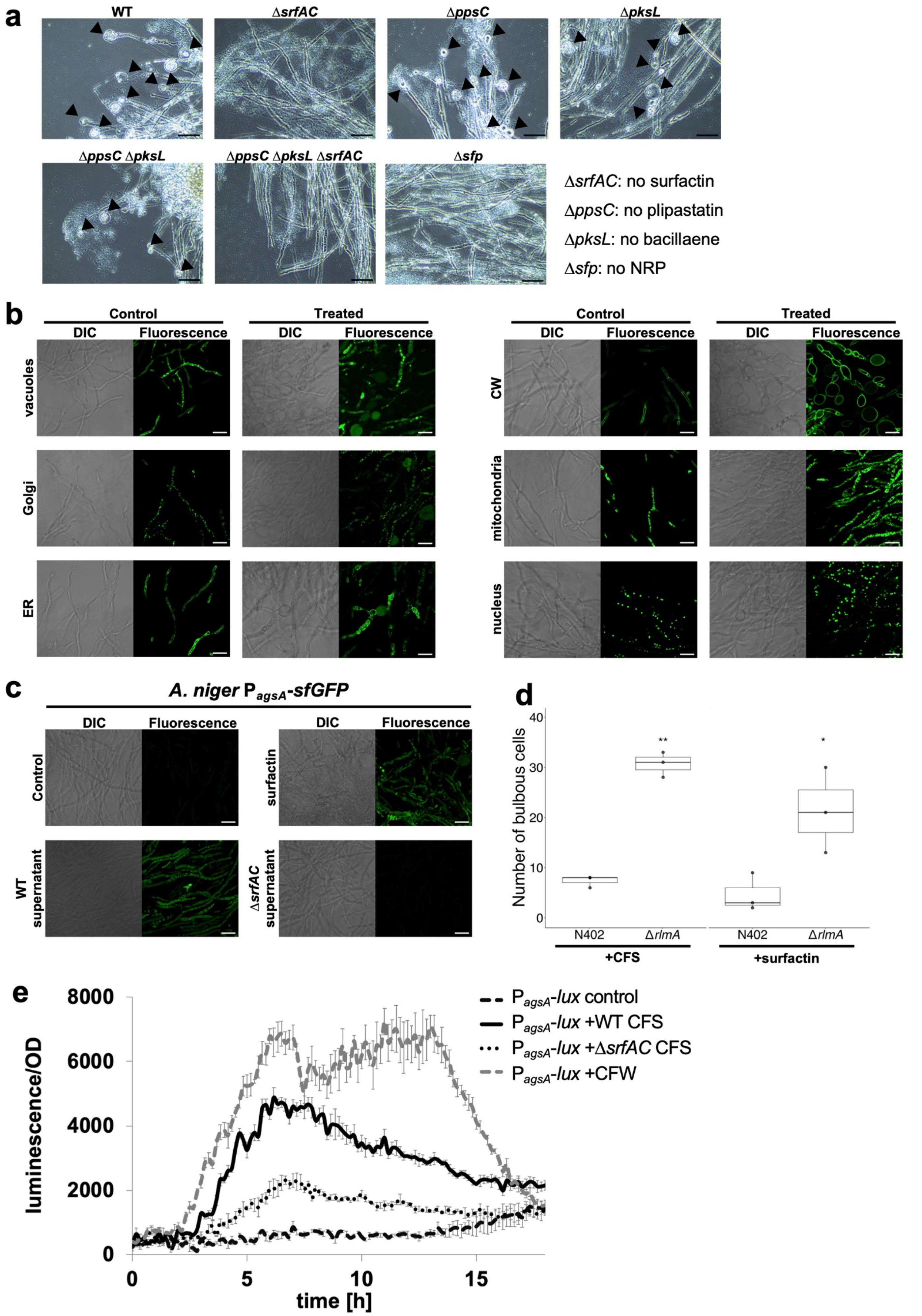
a
WT
ΔsrfAC
ΔppsC
ΔpksL
ΔppsC ΔpksL
ΔppsC ΔpksL ΔsrfAC
Δsfp
ΔsrfAC: no surfactin
ΔppsC: no plipastatin
ΔpksL: no bacillaene
Δsfp: no NRP
b
Control
Treated
DIC
Fluorescence
vacuoles
Golgi
ER
CW
mitochondria
nucleus
c
A. niger P agsA -sfGFP
DIC
Fluorescence
Control
surfactin
WT supernatant
ΔsrfAC supernatant
d
Number of bulbous cells
40
30
20
10
0
**
*
N402
ΔrlmA
N402
ΔrlmA
+CFS
+surfactin
e
luminescence/OD
8000
6000
4000
2000
0
0
5
10
15
time [h]
P agsA -lux control
P agsA -lux +WT CFS
P agsA -lux +ΔsrfAC CFS
P agsA -lux +CFW

Supplementary figure 6 | Influence of surfactin on fungal hyphae. a, Microscopy visualisation of bulging fungal hyphae with wild type (WT) and various mutants, including strain lacking surfactin (Δ*srfAC*), plipastatin (Δ*ppsC*), bacillaene (Δ*pksL*), plipastatin and bacillaene (Δ*ppsC* Δ*pksL*), plipastatin, bacillaene, and surfactin (Δ*ppsC* Δ*pksL* Δ*srfAC*), or all non-ribosomal peptides (Δ*sfp*). Scale bar = 20 μm. **b,** DIC (left) and green fluorescence (right) imaging of the *A. niger* MA23.1.1 strain for vacuoles (P_{gpdA}-CpyA::eGFP-T*trpC*); Ren1.10 strain for Golgi (P_{gmtA}-eYFP:: GMTA-T*gmtA*), MA141.1 strain for endoplasmic reticulum, ER (P_{gpdA}-*glaA*:: sGFP-HDEL-T*trpC*); AR0#11 strain for cell wall, CW (P_{gpdA}-*glaA*::sGFP-T*trpC*); BN38.9 strain for mitochondria (P_{gpdA}-CitA::eGFP-T*trpC*); and MA26.1 strain for nucleus (P_{gpdA}-H2B::eGFP-T*trpC*) in the absence (Control) or presence (Treated) of bacterial cell free supernatant. Scale bar = 20 μm. **c,** DIC (left) and green fluorescence (right) imaging of the *A. niger* JvD1.1 strain carrying P_{agsA}-eGFP-T*trpC* for detection of *agsA* gene expression in the absence (control) and presence of cell-free WT supernatant, 20 μg/ml surfactin, and cell-free Δ*srfAC* supernatant. Scale bar = 20 μm. Images in panel a, b, and c are representative examples from three experiments. **d,** Number of bulbous cells by the wild-type N402 and Δ*rlmA* mutant *A. niger* in the presence of cell-free WT supernatant (CFS) or 20 μg/ml surfactin. Student's t-test with Bonferroni-Holm correction was performed (*p_{adjust} <0.05, **p_{adjust} <0.01, n=3). **e,** Luminescence reporter assay using *A. niger* strains MA297.3 containing $P_{agsA(3\times\text{RlmA box})}$ before the promoter-less luciferase. Cultures were treated with LB medium (black dashed line), cell-free WT supernatant (CFS, solid line), cell-free Δ*srfAC* supernatant (Δ*srfAC* CSF, dotted line), or Calcofluor White (CFW, grey dashed line).

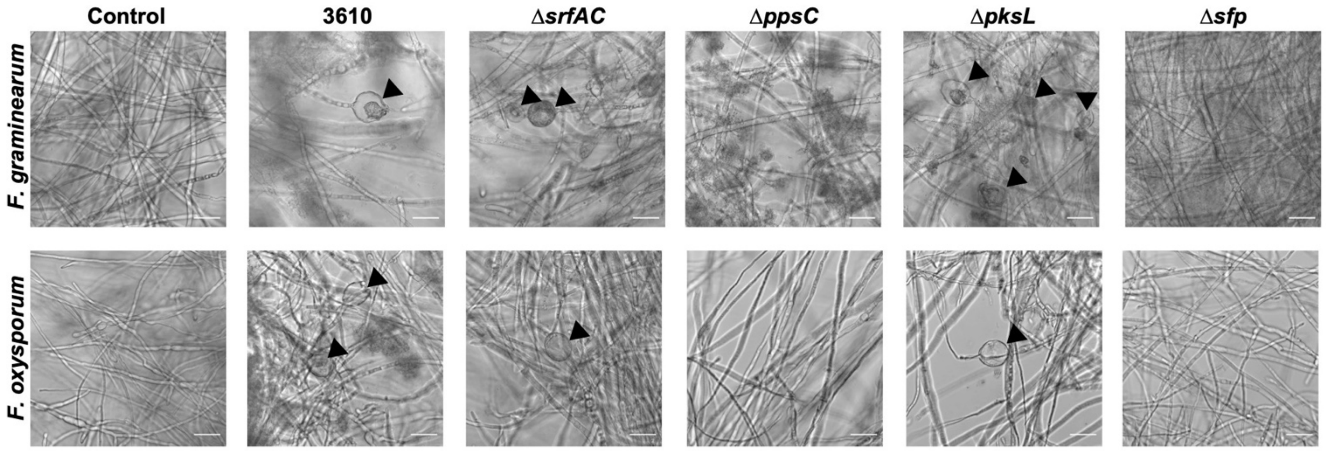

Supplementary figure 7 | Influence of plipastatin on hyphae of Fusarium species. Microscopy visualisation of bulging Fusarium hyphae with wild type (WT) and various mutants, including strain lacking surfactin (Δ*srfAC*), plipastatin (Δ*ppsC*), bacillaene (Δ*pksL*), or all non-ribosomal peptides (Δ*sfp*). Scale bar = 25 μm. Images are representative examples from three photos taken for each culture.

Supplementary Note 1

CoEvo3 isolates respond to fungal volatiles, resulting in reduced spreading. While isolating the CoEvo strains, increased spreading over the agar surface was observed compared with the ancestor strain. Intriguingly, while CoEvo2 isolates displayed increased spreading on 1% agar medium regardless of the presence of the fungus, the CoEvo3 clone was only able to expand on the agar surface in the absence of the fungus, even if separated from the fungus by a plastic barrier allowing only volatile-mediated interaction (Supplementary Fig. 2a). The recreated *degS*^{A193V} strain phenocopied the influence of *A. niger* volatiles on colony expansion (Supplementary Fig. 2b). Therefore, for the CoEvo3 strain, the volatilomes of single and separated co-cultures were determined at day 3 and day 7 after bacterial inoculation (see experimental setup in Supplementary Fig. 2c). Volatilomes contained dimethyl disulphide (DMS), pyrazine and 1-pentyne when *A. niger* was present but not when *B. subtilis* was cultured alone (for a full list see Supplementary Dataset 2). Supplementing either the pure compounds or the mixture of these volatile organic compounds (VOCs) reduced colony spreading of CoEvo3 (Supplementary Fig. 2d, one-way ANOVA, $F = 27.04$, $p < 0.001$, Tukey's HSD), demonstrating the volatile-mediated influence of *A. niger* on specific evolved isolates of *B. subtilis*. However, since these experiments were performed using a CoEvo3 isolate, we cannot exclude the influence of these VOCs on the ancestor strain, which did not display enhanced surface spreading under these conditions. Further experiments are needed to reveal the direct influence of the identified VOCs on the growth, physiology, or differentiation of *B. subtilis*.

Supplementary Table 1 for strains, plasmids and oligos

B. subtilis strains	Genotype, description	Reference
NCIB 3610	undomesticated wild type strain	[1,2]
DK1042	NCIB 3610, but *comI*^{Q12L} (naturally competent)	[3]
168 *degU*	*trpC* Δ*degU*::Km^{R}	[4]
TB742	DK1042 Δ*degU*::Km^{R}	This work
TB938	DK1042 *degU*^{S202G}	This work
TB939	DK1042 *degS*^{A193V}	This work
DS4085	NCIB 3610 Δ*pksL*::Cm^{R}	[5]
DS4114	NCIB 3610 Δ*ppsC*::Tet^{R}	[5]
DS1122	NCIB 3610 *srfAC*::Tn10 Spec^{R}	[6]
DS3337	NCIB 3610 Δ*sfp*::Mls^{R}	[7]
DS4113	NCIB 3610 Δ*ppsC*::Tet^{R} Δ*pksL*::Cm^{R}	[5]
DS4124	NCIB 3610 Δ*ppsC*::Tet^{R} Δ*pksL*::Cm^{R} *srfAC*::Tn10 Spec^{R}	[5]
A. niger strains	Genotype	Reference
N402	wild-type fungal strain	[8]
MA297.3	N402 $P_{agsA(3\times \mathrm{RlmA\ box})}$-m*luc*-T*trpC-pyrG***	This work
MA584.2	N402 $P_{agsA(\mathrm{RlmA\ box\ mutated})}$-m*luc*-T*trpC-pyrG***	This work
Δ*rlmA*	N402 Δ*rlmA*::hyg^{R}	[9]
AR0#11	N402 P_{gpdA}-*glaA*::sGFP-T*trpC*	[10]
MA141.1	N402 P_{gpdA}-*glaA*:: sGFP-HDEL-T*trpC*	[11]
Ren1.10	N402 P_{gmtA}-eYFP:: GMTA-T*gmtA*	[11]
MA23.1.1	N402 P_{gpdA}-CpyA::eGFP-T*trpC*	[12]
FG7	N402 P_{synA}-eGFP::SynA-T*synA*	[13]
BN38.9	N402 P_{gpdA}-CitA::eGFP-T*trpC*	[14]
MA26.1	N402 P_{gpdA}-H2B::eGFP-T*trpC*	[12]
JvD1.1	N402 P_{agsA}-eGFP-T*trpC*	[15]
AR19.1	M402 P_{gpdA}-sGFP-T*trpC*	[16]
MA824.1	N402 *oahA*::*AOpyrG*	This work
Fungal strains		
A. awamori	FSU 11418	JMRC
A. brasiliensis	FSU 35902 (DSM 1988)	JMRC
A. tubingiensis	FSU 11408	JMRC
A. nidulans	HKI G034	JMRC
F. graminearum	IBT 41925	IBT
F. oxysporum	IBT 40872	IBT
JMRC: Jena Microbial Resource Collection at Leibniz Institute for Natural Product Research and Infection Biology Hans Knöll Institute, Jena, Germany (https://www.leibniz-hki.de/en/jena-microbial-resource-collection.html)<br>IBT: IBT Culture Collection at DTU Bioengineering, Kongens Lyngby, Denmark (https://www.bioengineering.dtu.dk/research/strain-collections/ibt-culture-collection-of-fungi)		
Plasmids	description	Source
pMiniMad	*ori*^{BsTs} *Amp*^{R} *Mls*^{R}	[17]
pTB693	pMiniMad with *degU*^{S202G}	This work

pTB694	pMiniMad with *degS*^{A193V}	This work
pMA334	vector with *pyrG* flanking regions	[18]
pBN008	vector with P_{agsA}(0.55-kb-rlm2add)-*uidA-pyrG*	[9]
pVG4.1	vector with m*luc*-T*trpC*	[19]
pMA348	pMA334 with $P_{agsA(3\times RlmA\text{-}box)}$-m*luc*-T*trpC-pyrG***	This work
pMA370	pMA334 with $P_{agsA(RlmA\text{-}box\ mutated)}$-m*luc*-T*trpC-pyrG***	This work

Oligos		sequence	gene targeted
oAR23	*Nco*I	ATCCATGGTGGCGGCTGAGAAGTCGTCG	*degU* in CoEvo2
oAR24	*Bam*HI	GCGGATCCAAGAGGTTATCTGCTGAAAG	*degU* in CoEvo2
oAR30	*Sal*I	CCGTCGACTTGGCGATAAACTTGAAGTG	*degS* in CoEvo3
oAR41	*Bam*HI	ATGGATCCTGAAGAGCGCAACCTCAAAC	*degS* in CoEvo3
oAR25		AGACTTGCCAAGCTCTTC	*degU*
oAR26		GCTTGTAGAGCTGTATCC	*degU*
oAR31		TCAGGTCGAACCCTTTAC	*degS*
oAR32		AACAGCTGGTCGAAGAAC	*degS*
oAR27		TCCTCTGGCCATTGCTCTG	pMiniMad MCS
oAR28		CGAAGTTAGGCTGGTAAG	pMiniMad MCS
PagsAP1f-NotI		GCGGCCGCTCTAGAACTAGT	
TtrpCP2r-NotI		AAGGAAAAAAGCGGCCGCTCTAGAAAGAAGGATTACCTC	
PagsAP4f-NotI		AAGGAAAAAAGCGGCCGCCTGCAGTAGTGGCGGCTGCTTC	
PagsA-AF-R-mut-RlmA2		CTCGGTGGTCGCCGCCGAGAAACGTCATATCAGGATAGC	
PagsA-AF-F-mut-RlmA1		ATATGACGTTTCTCGGCGGCGACCACCGAGAGTAGAGAATGA	
PagsAP2r		CTCGATCTTTCTGCGACCCATGATGGCAAGCGGCGTGTGGTA	
oahAP1f		ACCATCACCGTAGAGCAGGAC	
oahAP11r		CAATTCCAGC AGCGGCTTGC GAGAAAAGCA CAAGGTATA	
oahAP3f		ACACGGCACA ATTATCCATC GGATTCTTAA TTCCCTGGTT GTTTTG	
oahAP4r		CGGACGACGG AGTTTGAATC	
AOpyrGP12f		AAGCCGCTGCTGGAATTG	
AOpyrGP15r		CCGGTAGCCAAAGATCCCTT	
AOpyrGP13r		CGATGGATAATTGTGCCGTGT	
AOpyrGP14f		ATTGACCTACAGCGCACGC	
oBK7		CCGAGTACAAGGARGCCTTC	CaM
oBK8		CCGATRGAGGTCATRACGTGG	CaM

Supplementary References

1. Branda, S. S., González-Pastor, J. E., Ben-Yehuda, S., Losick, R. & Kolter, R. Fruiting body formation by *Bacillus subtilis*. *Proc Natl Acad Sci U S A* **98**, 11621–11626 (2001).
2. Zeigler, D. R. *et al.* The origins of 168, W23, and other *Bacillus subtilis* legacy strains. *J Bacteriol* **190**, 6983–6995 (2008).
3. Konkol, M. A., Blair, K. M. & Kearns, D. B. Plasmid-encoded comI inhibits competence in the ancestral 3610 strain of *Bacillus subtilis*. *J Bacteriol* **195**, 4085–4093 (2013).
4. Kovács, Á. T. & Kuipers, O. P. Rok regulates *yuaB* expression during architecturally complex colony development of *Bacillus subtilis* 168. *J Bacteriol* **193**, 998–1002 (2011).
5. Müller, S. *et al.* Bacillaene and sporulation protect *Bacillus subtilis* from predation by *Myxococcus xanthus*. *Appl Environ Microbiol* **80**, 5603–5610 (2014).

6. Chen, R., Guttenplan, S. B., Blair, K. M. & Kearns, D. B. Role of the σD-dependent autolysins in *Bacillus subtilis* population heterogeneity. *J Bacteriol* **191**, 5775–5784 (2009).
7. Patrick, J. E. & Kearns, D. B. Laboratory strains of *Bacillus subtilis* do not exhibit swarming motility. *J Bacteriol* **191**, 7129–7133 (2009).
8. Bos, C. J. *et al.* Genetic analysis and the construction of master strains for assignment of genes to six linkage groups in *Aspergillus niger*. *Curr Genet* **14**, 437–443 (1988).
9. Damveld, R. A. *et al.* The *Aspergillus niger* MADS-box transcription factor RlmA is required for cell wall reinforcement in response to cell wall stress. *Mol Microbiol* **58**, 305–319 (2005).
10. Gordon, C. L. *et al.* Glucoamylase::green fluorescent protein fusions to monitor protein secretion in *Aspergillus niger*. *Microbiology (N Y)* **146**, 415–426 (2000).
11. Carvalho, N. D. S. P. *et al.* Functional YFP-tagging of the essential GDP-mannose transporter reveals an important role for the secretion related small GTPase SrgC protein in maintenance of Golgi bodies in *Aspergillus niger*. *Fungal Biol* **115**, 253–264 (2011).
12. Weenink, X. O. Protein secretion in the filamentous fungus *Aspergillus niger*. (Leiden University, 2008).
13. Kwon, M. J. *et al.* Molecular genetic analysis of vesicular transport in *Aspergillus niger* reveals partial conservation of the molecular mechanism of exocytosis in fungi. *Microbiology (N Y)* **160**, 316–329 (2014).
14. Nitsche, B. M., Burggraaf-Van Welzen, A. M., Lamers, G., Meyer, V. & Ram, A. F. J. Autophagy promotes survival in aging submerged cultures of the filamentous fungus *Aspergillus niger*. *Appl Microbiol Biotechnol* **97**, 8205–8218 (2013).
15. Meyer, V. *et al.* Survival in the presence of antifungals: Genome-wide expression profiling of *Aspergillus niger* in response to sublethal concentrations of caspofungin and fenpropimorph. *Journal of Biological Chemistry* **282**, 32935–32948 (2007).
16. Vinck, A. *et al.* Hyphal differentiation in the exploring mycelium of *Aspergillus niger*. *Mol Microbiol* **58**, 693–699 (2005).
17. Patrick, J. E. & Kearns, D. B. MinJ (YvjD) is a topological determinant of cell division in *Bacillus subtilis*. *Mol Microbiol* **70**, 1166–1179 (2008).
18. Arentshorst, M., Lagendijk, E. L. & Ram, A. F. A new vector for efficient gene targeting to the *pyrG* locus in *Aspergillus niger*. *Fungal Biol Biotechnol* **2**, 2 (2015).
19. Meyer, V. *et al.* Fungal gene expression on demand: An inducible, tunable, and metabolism-independent expression system for *Aspergillus niger*. *Appl Environ Microbiol* **77**, 2975–2983 (2011).
