# Supplementary material for: Enhanced surface colonisation and competition during bacterial adaptation to a fungus
Source: Nat Commun. 2024 May 27;15:4486. doi: 10.1038/s41467-024-48812-1 (PMC11130161; doi:10.1038/s41467-024-48812-1)
Supplement: Supplementary file 3 — Description of Additional Supplementary Files [file 41467_2024_48812_MOESM3_ESM.pdf]

Description of Additional Supplementary Files:

Supplementary Dataset 1: Final mutation set of CoEvo lineages. The table shows final mutation set of all sequenced populations. Type of mutations: SNP, single nucleotide polymorphism, DEL, deletion, INS, insertion.

Supplementary Dataset 2: Dataset of volatiles at 3 and 7 days. Tentatively identified volatile organic compounds (VOCs) produced by A. niger N402 and B. subtilis CoEvo3 in mono- and co-culture on LB-agar.

Supplementary Dataset 3: Sequences of PCR-amplified calmodulin gene fragment of the fungal strains obtained from the Jena Microbial Resource Collection (JMRC).

Supplementary Movie 1: Time lapse video of the ancestor B. subtilis (middle) and A. niger N402 (streaked above and below the bacterial spot a day before) on LB agar medium in a 45 mm petri dish.

Supplementary Movie 2: Time lapse video of the B. subtilis CoEvo2 isolate (middle) and A. niger N402 (streaked above and below the bacterial spot a day before) on LB agar medium in a 45 mm petri dish.
