# Supplementary material for: Enhanced surface colonisation and competition during bacterial adaptation to a fungus
Source: Nat Commun. 2024 May 27;15:4486. doi: 10.1038/s41467-024-48812-1 (PMC11130161; doi:10.1038/s41467-024-48812-1)
Supplement: Supplementary file 9 — Reporting Summary [file 41467_2024_48812_MOESM9_ESM.pdf]

nature portfolio

Corresponding author(s): Ákos T. kovács

Last updated by author(s): Apr 29, 2024

# Reporting Summary

Nature Portfolio wishes to improve the reproducibility of the work that we publish. This form provides structure for consistency and transparency in reporting. For further information on Nature Portfolio policies, see our Editorial Policies and the Editorial Policy Checklist.

## Statistics

For all statistical analyses, confirm that the following items are present in the figure legend, table legend, main text, or Methods section.

| n/a | Confirmed | |
|---|---|---|
| ☐ | ☒ | The exact sample size (*n*) for each experimental group/condition, given as a discrete number and unit of measurement |
| ☐ | ☒ | A statement on whether measurements were taken from distinct samples or whether the same sample was measured repeatedly |
| ☐ | ☒ | The statistical test(s) used AND whether they are one- or two-sided<br>*Only common tests should be described solely by name; describe more complex techniques in the Methods section.* |
| ☒ | ☐ | A description of all covariates tested |
| ☒ | ☐ | A description of any assumptions or corrections, such as tests of normality and adjustment for multiple comparisons |
| ☐ | ☒ | A full description of the statistical parameters including central tendency (e.g. means) or other basic estimates (e.g. regression coefficient) AND variation (e.g. standard deviation) or associated estimates of uncertainty (e.g. confidence intervals) |
| ☒ | ☐ | For null hypothesis testing, the test statistic (e.g. *F*, *t*, *r*) with confidence intervals, effect sizes, degrees of freedom and *P* value noted<br>*Give P values as exact values whenever suitable.* |
| ☒ | ☐ | For Bayesian analysis, information on the choice of priors and Markov chain Monte Carlo settings |
| ☒ | ☐ | For hierarchical and complex designs, identification of the appropriate level for tests and full reporting of outcomes |
| ☒ | ☐ | Estimates of effect sizes (e.g. Cohen's *d*, Pearson's *r*), indicating how they were calculated |

*Our web collection on statistics for biologists contains articles on many of the points above.*

## Software and code

Policy information about availability of computer code

| | |
|---|---|
| Data collection | No data from other source was used |
| Data analysis | MetaboAnalyst V3.0 (https://www.metaboanalyst.ca/home.xhtml); R environment for statistical computing and graphics 4.3.1 (The R Foundation) |

For manuscripts utilizing custom algorithms or software that are central to the research but not yet described in published literature, software must be made available to editors and reviewers. We strongly encourage code deposition in a community repository (e.g. GitHub). See the Nature Portfolio guidelines for submitting code & software for further information.

## Data

Policy information about availability of data

All manuscripts must include a data availability statement. This statement should provide the following information, where applicable:

- Accession codes, unique identifiers, or web links for publicly available datasets
- A description of any restrictions on data availability
- For clinical datasets or third party data, please ensure that the statement adheres to our policy

The following statement included in manuscript:
Population sequencing data have been deposited in the CNGB Sequence Archive (CNSA) 84 of the China National GeneBank DataBase (CNGBdb) 85 under accession number CNP0003923 (https://db.cngb.org/search/project/CNP0003923/). Data for the DK1042 ancestor strain are available under CNP0002416 (https://

db.cngb.org/search/project/CNP0002416/). Isolate sequencing data have been deposited at the NCBI Sequence Read Archive (SRA) database under BioProject accession numbers PRJNA625867 (ancestor, https://www.ncbi.nlm.nih.gov/bioproject/PRJNA625867) and PRJNA926387 (evolved clones, https://www.ncbi.nlm.nih.gov/bioproject/PRJNA926387). VOC data available on Metabolomics Workbench as project PR001621 (http://dx.doi.org/10.21228/M85M6X). Data points are all included in Supplementary Datasets. All other data generated and analysed during this study are either available in Source Data or can be requested from the corresponding author.

## Research involving human participants, their data, or biological material

Policy information about studies with human participants or human data. See also policy information about sex, gender (identity/presentation), and sexual orientation and race, ethnicity and racism.

| | |
|---|---|
| Reporting on sex and gender | N/A |
| Reporting on race, ethnicity, or other socially relevant groupings | N/A |
| Population characteristics | N/A |
| Recruitment | N/A |
| Ethics oversight | N/A |

Note that full information on the approval of the study protocol must also be provided in the manuscript.

# Field-specific reporting

Please select the one below that is the best fit for your research. If you are not sure, read the appropriate sections before making your selection.

☒ Life sciences ☐ Behavioural & social sciences ☐ Ecological, evolutionary & environmental sciences

For a reference copy of the document with all sections, see nature.com/documents/nr-reporting-summary-flat.pdf

# Life sciences study design

All studies must disclose on these points even when the disclosure is negative.

| | |
|---|---|
| Sample size | No sample size calculations were used before obtaining data. Such was chosen based on experience and knowledge on expected biological variability (which cannot be controlled by the experimenter) and accuracy of the experimental methods used. At least 3-5 biological replicates were used for all experiments, the sample size used to generate quantitative data was assessed to see the distribution of the data points. |
| Data exclusions | No data were excluded from the analyses. |
| Replication | Each experiments, except the original experimental evolution setup, were replicated on different days to ensure repeatability. To ensure reliable replication of experiments described in this manuscript the culture stocks were securely stored deep-frozen, each experiment was initiated from the same culture stock following the methods exactly as described in this manuscript. All experiments contained biological replicates, vast majority of the experiments were performed multiple times, and several key experiments were perfomed by indendent experimenters or even in research labs at different countries (Germany, Denmark and the Netherlands. All attempts at experiments replications were successful. |
| Randomization | Randomization was not relevant in this study. Comparisons were not performed on individual organisms (with high level of individual variability) but on large populations of microbes that were independently initiated from small number of cells. Growth conditions were strictly controlled by the experimenter and identical for each population. |
| Blinding | Blinding was not considered in this study, bacuase methods used in the assessment of the results were objective. |

# Reporting for specific materials, systems and methods

We require information from authors about some types of materials, experimental systems and methods used in many studies. Here, indicate whether each material, system or method listed is relevant to your study. If you are not sure if a list item applies to your research, read the appropriate section before selecting a response.

## Materials & experimental systems

| n/a | Involved in the study | |
|---|---|---|
| ☒ | ☐ | Antibodies |
| ☒ | ☐ | Eukaryotic cell lines |
| ☒ | ☐ | Palaeontology and archaeology |
| ☒ | ☐ | Animals and other organisms |
| ☒ | ☐ | Clinical data |
| ☒ | ☐ | Dual use research of concern |
| ☒ | ☐ | Plants |

## Methods

| n/a | Involved in the study | |
|---|---|---|
| ☒ | ☐ | ChIP-seq |
| ☒ | ☐ | Flow cytometry |
| ☒ | ☐ | MRI-based neuroimaging |

## Plants

| | |
|---|---|
| Seed stocks | N/A |
| Novel plant genotypes | N/A |
| Authentication | N/A |
